# Supplementation of Chlorogenic Acid Alleviates the Effects of H_2_O_2_-Induced Oxidative Stress on Laying Performance, Egg Quality, Antioxidant Capacity, Hepatic Inflammation, Mitochondrial Dysfunction, and Lipid Accumulation in Laying Hens

**DOI:** 10.3390/antiox13111303

**Published:** 2024-10-27

**Authors:** Haitong Zhao, Zhuang Li, Yue Sun, Ming Yan, Yingjie Wang, Yurong Li, Yeshun Zhang, Mingkun Zhu

**Affiliations:** 1 Jiangsu Key Laboratory of Sericultural Biology and Animal Biotechnology, School of Biotechnology, Jiangsu University of Science and Technology, Zhenjiang 212100, China; 231211802118@stu.just.edu.cn (H.Z.); 222211801124@stu.just.edu.cn (Z.L.); 231211802111@stu.just.edu.cn (Y.S.); 221211802117@stu.just.edu.cn (M.Y.); yjwang@just.edu.cn (Y.W.); yrli@just.edu.cn (Y.L.); zyssri@just.edu.cn (Y.Z.); 2 Key Laboratory of Silkworm and Mulberry Genetic Improvement, Ministry of Agriculture and Rural Affairs, The Sericultural Research Institute, Chinese Academy of Agricultural Sciences, Zhenjiang 212100, China

**Keywords:** chlorogenic acid, hydrogen peroxide, oxidative stress, liver, lipid metabolism, laying hen

## Abstract

This research examined the impact of chlorogenic acid (CGA) on laying performance, antioxidant capacity, egg quality, hepatic inflammation, mitochondrial function, and lipid metabolism in hens subjected to hydrogen peroxide (H_2_O_2_)-induced oxidative stress (OS). Three hundred sixty healthy 43-wk-old Hy-Line brown hens were randomly assigned to six treatments: a basal diet + 0 (control and H_2_O_2_), 600 (600 mg/kg CGA and 600 mg/kg CGA + H_2_O_2_), and 800 (800 mg/kg CGA and 800 mg/kg CGA + H_2_O_2_) mg/kg CGA for 84 d. On the 64th and 78th days of the trial, hens in groups H_2_O_2_, 600 mg/kg CGA + H_2_O_2_, and 800 mg/kg CGA + H_2_O_2_ were injected intraperitoneally with 10% H_2_O_2_. The results demonstrated that 600 and 800 mg/kg CGA significantly improved the egg production rate (EPR) and egg quality and reduced lipid peroxidation compared to the control group. The 800 mg/kg CGA showed greater improvements in the EPR and average egg weight (AEW) compared to the 600 mg/kg dose. Conversely, H_2_O_2_ exposure significantly decreased the EPR, AEW, and egg quality and increased feed conversion rate and average daily feed intake. H_2_O_2_ exposure significantly decreased serum T-AOC and increased serum MDA levels while reducing hepatic T-SOD, GSH-Px, and CAT activities. Meanwhile, H_2_O_2_ exposure significantly elevated liver reactive oxygen species levels, pathological damage, and *NF-κB*, *TNFα*, and *IL-1β* gene expression. Additionally, H_2_O_2_ treatment disrupted hepatocyte mitochondrial structure and significantly increased the expression of VDAC1 protein, and *IP3R*, *GRP75*, *MCU*, *Fis1*, and *MFF* genes, while downregulating the expression of MFN2 protein and *PGC1α* gene. Oil Red O staining demonstrated that H_2_O_2_ induced significant lipid accumulation in hepatocytes. Concurrently, H_2_O_2_ significantly increased serum triglycerides, total cholesterol, and liver triglycerides levels while decreasing serum hepatic lipase activity. This was primarily attributed to the significant upregulation of liver *SREBP1*, *FASN*, and *ACC* genes and the downregulation of the liver *CPT1* gene induced by H_2_O_2_. Furthermore, CGA pretreatment effectively prevented the degeneration in laying performance and egg quality, as well as OS, liver inflammation, pathological damage, and mitochondrial dysfunction induced by H_2_O_2_. CGA inhibited H_2_O_2_-induced hepatic lipid accumulation by upregulating fatty acid oxidation-related gene expression and downregulating fatty acid synthesis-related gene expression. These findings indicate that the dietary addition of 800 mg/kg of CGA is the optimum supplementation dose. CGA can enhance laying performance and egg quality while alleviating OS, hepatic inflammation, mitochondrial dysfunction, and lipid accumulation in H_2_O_2_-challenged laying hens.

## 1. Introduction

In the practical setting of production, intensively raised hens are frequently subjected to a range of oxidative triggers, including increased ambient temperatures, toxic metals, fungal toxins, pathogens, and other detrimental agents, resulting in the onset of oxidative stress (OS) and consequent harm [1,2,3,4]. The impact of oxidative damage as a universal mechanism in poultry and livestock has been recognized as a crucial element leading to the detrimental effects of multiple stressors [5,6]. Prior investigations have consistently demonstrated that OS arising from diverse origins can have adverse effects on laying performance, deteriorate egg quality, overpower antioxidant mechanisms, undermine the structure of the intestinal mucosa, trigger systemic or localized inflammation, and disrupt the equilibrium of the gut microbiota in laying hens [7,8].

OS exerts a critical role in liver disease [9]. The increase in OS resulting from different factors causes hepatic lipid accumulation, whereas a decrease in OS exerts a lipid-lowering action on hepatocytes [10,11,12]. Abnormal accumulation of saturated fats such as triglycerides in the liver is linked to the onset and progression of non-alcoholic fatty liver disease [13]. This abnormal accumulation can further result in mitochondrial dysfunction and overproduction of reactive oxygen species (ROS) [14]. In laying hens, the liver is responsible for most lipid synthesis, including cholesterol and triglycerides (TGs). These lipids are subsequently transported via lipoproteins to the ovarian follicles, where they are integral to the production of egg yolk [15]. When the synthesis of fat surpasses the production of lipoproteins responsible for its transport, an accumulation of TGs occurs, leading to hepatic steatosis development [16]. Under modern intensive, large-scale farming models, this elevates the risk of fatty liver development in layers due to long-term positive energy state and insufficient physical exercise [17]. Meanwhile, OS caused by different environmental stress (heat stress, heavy metal, etc.) also significantly contributes to hepatic lipid accumulation in layers [18,19]. It has been proven that supplementation of substances with antioxidant activity in the diet can regulate lipid metabolism by improving the body’s redox state [18,20].

Chlorogenic acid (CGA), a naturally occurring polyphenolic compound also known as coffee tannic acid and coffee ellagic acid, is found primarily in coffee, tea, vegetables, and fruits [21]. It has been shown to safeguard cell survival by reducing excessive production of NO and ROS and inhibiting pro-apoptotic signaling, thereby preventing toxicity caused by different classes of substances, including toxins, drugs, metals, pesticides, etc. [22,23,24]. Zha et al. showed that adding chlorogenic acid to broiler diets alleviated diquat-induced OS and hepatic inflammation [25]. Bi et al. demonstrated that dietary supplementation of CGA improved egg quality and enhanced antioxidant capacity in aged hens [26]. Additionally, CGA has proven effective in inhibiting weight gain and has exhibited beneficial lipid-lowering properties. In particular, CGA treatment reduces cholesterol and TG concentrations in both the serum and liver of mice fed a high-fat diet [27,28]. Hence, we hypothesized that CGA exerts beneficial effects to resist the hydrogen peroxide (H_2_O_2_)-induced OS and liver damage in hens by maintaining redox status and mitochondrial function, inhibiting inflammation, and promoting hepatic lipid metabolism. This research was conducted to assess the safeguarding effects of CGA on laying hens exposed to OS induced by an H_2_O_2_ challenge. The results will offer a reference and theoretical foundation for alleviating OS and the appropriate use of CGA in hens’ diets.

## 2. Materials and Methods

### 2.1. Animals and Experimental Design

Three hundred sixty healthy 43-week-old Hyline brown hens with similar body weight and laying rates were sourced from a Zhenjiang farm and randomly assigned into six groups with six replicates of 10 hens in each (two hens per cage). The hens were fed a basal diet + 0 (control and H_2_O_2_ groups), 600 (600 mg/kg CGA and 600 mg/kg CGA + H_2_O_2_ groups), and 800 (800 mg/kg CGA and 800 mg/kg CGA + H_2_O_2_ groups) mg/kg CGA (powder, 98% CGA, Lvyouran Biotechnology (Xi’an) Co., Ltd., Xi’an, China). The different doses of CGA powder were thoroughly mixed with the basal diet. The experiment lasted 14 weeks, consisting of a 2-week pre-feeding phase (in which the basal diet was provided), followed by a 12-week experimental period. Hens in groups H_2_O_2_, 600 mg/kg CGA + H_2_O_2_, and 800 mg/kg CGA + H_2_O_2_ received intraperitoneal injections of 10% H_2_O_2_ (2.96 mmol H_2_O_2_/kg body weight) on the first day of weeks 10 and 12, respectively. Hens are housed in advanced three-tiered steeped egg cages used in the experiment (individual cage size: 47 cm × 35 cm × 37 cm), equipped with a fully automatic belt-type manure removal system. The flooring of the chicken house is flat, and the temperature is controlled between 23 and 26 °C, with humidity maintained at 65–75%. The lighting period was set at 16 h per day. The basal diets were prepared in the laboratory, and the formulation is listed in Table 1.

### 2.2. Sample Collection and Measurement

Daily measurements of egg production and egg weight were documented for every replicate, while feed intake was evaluated for each replicate every two weeks. These measurements were used to calculate the egg production rate (EPR), average daily feed intake (ADFI), average egg weight (AEW), and feed conversion ratio (FCR). Thirty eggs from every treatment were obtained in the 12th week for egg quality assessment using a digital egg tester (DET-6000, Nabel Co., Ltd., Kyoto, Japan).

Following the completion of the trial, 12 hens (2 hens per replicate) from each treatment were chosen to undergo twelve hours of fasting (free access to water). Blood samples were then collected via venipuncture of the wing vein and centrifuged at 3000 rpm for 10 min to isolate serum for subsequent antioxidant and lipid metabolism analysis. Finally, the chickens were euthanized by CO_2_ inhalation, and the liver tissue was immediately removed and divided into six portions. Four parts of the liver were promptly cryopreserved at −80 °C for antioxidant and molecular analysis, as well as Oil Red O and ROS staining. The other two portions of the liver were sampled in 4% paraformaldehyde and glutaraldehyde, respectively, for histopathological and mitochondrial structure analysis.

### 2.3. Serum and Liver Antioxidant Enzyme Detection

Each 0.1 g liver sample was homogenized in 900 µL of cold PBS to formulate a ten percent tissue homogenate. After centrifugation at 2500× *g* for 10 min at 4 °C, the supernatant was gathered for antioxidant ability assessment. The concentrations of H_2_O_2_ and malondialdehyde (MDA), along with the activities of total antioxidant capacity (T-AOC), total superoxide dismutase (T-SOD), glutathione peroxidase (GSH-Px), and catalase (CAT) were measured utilizing assay kits procured from Nanjing Jiancheng Bioengineering Institute (Nanjing, China). We quantified the homogenate protein concentration utilizing a BCA protein assay kit from Beyotime (Shanghai, China).

### 2.4. Serum and Liver Lipid Assay

The lipoprotein lipase (LPL) and hepatic lipase (HL) activities, and the TGs, total cholesterol (T-CHO), and low-density lipoprotein cholesterol (LDL-C) levels in the serum and/or liver were quantified utilizing assay kits procured from Nanjing Jiancheng Bioengineering Institute.

### 2.5. Hematoxylin and Eosin Staining

As previously outlined [19], liver samples were immersed in 4% paraformaldehyde for a minimum duration of 24 h and subsequently embedded in paraffin. The embedded samples were sectioned into 5-μm thick slices using a Leica RM2016 paraffin microtome (Leica, Germany). The tissue sections underwent dewaxing with xylene and were dehydrated through a graded series of ethanol solutions. Following staining with hematoxylin and eosin, the sections were imaged utilizing an OLYMPUS optical microscope (Tokyo, Japan).

### 2.6. Oil Red O Staining

As previously outlined [19], frozen liver sections (approximately 8 μm thick) were fixed in 4% paraformaldehyde for 15 min and then washed three times. After being air-dried, the slides were stained with Oil Red O reagent (G1015, Servicebio, Wuhan, China) for 10 min. They were subsequently washed with 60% isopropanol and counterstained with hematoxylin. The sections were imaged using an OLYMPUS optical microscope (Tokyo, Japan) after being sealed with glycerin gelatin (G1402, Servicebio, Wuhan, China).

### 2.7. ROS Staining

The frozen liver sections were defrosted at ambient temperature. Tissue boundaries were delineated using a histochemical pen, and a self-fluorescence quencher (G1221, Servicebio, Wuhan, China) was used for 5 min, followed by a 10 min rinse under running water. ROS dye solution (D7008, Sigma, Saint Louis, MO, USA) was then applied to the marked areas and maintained at 37 °C away from light for thirty minutes. Following rinsing, the DAPI dye solution (G1012, Servicebio, Wuhan, China) was applied and maintained at ambient temperature in the dark for ten minutes. Finally, an anti-fade mounting medium (G1401, Servicebio, Wuhan, China) was applied, and the samples were examined using a fluorescence microscope (Nikon, Tokyo, Japan).

### 2.8. Transmission Electron Microscope (TEM) Assay

Liver tissues were prefixed with 2.5% glutaraldehyde at 4 °C for 24 h and postfixed in 1% osmium tetroxide for two hours. Subsequently, the tissues underwent dehydration through graded ethanol and were embedded in epoxy resin. The embedding blocks were polymerized in a 60 °C oven for 48 h. Following staining with toluidine blue for localization, the blocks were sectioned into ultrathin slices with an ultramicrotome (Leica UC7, Germany). The sections were then sequentially stained in the dark with a 2% uranium acetate-saturated alcohol solution for eight minutes, followed by 2.6% lead citrate in the absence of carbon dioxide for 8 min before being dried overnight. Finally, a Hitachi-7800 TEM (Tokyo, Japan) was used for image capture.

### 2.9. Quantitative Real-Time PCR (qRT-PCR)

Total RNA was isolated from liver tissues with Trizol, and complementary DNA (cDNA) was synthesized using a reverse transcription kit (Takara RR047A, Dalian, China). QRT-PCR was run on the CFX96 Touch RT-PCR detection system (Bio-Rad, Hercules, CA, USA) using TB Green reagent (Takara RR420A, Dalian, China). The mRNA expression levels of inflammation-related genes (nuclear factor kappa B (*NF-κB*), tumor necrosis factor-alpha (*TNFα*), and interleukin-1 beta (*IL-1β*)), inositol 1,4,5-trisphosphate receptor (IP3R)/glucose-regulated protein 75 (GRP75)/volt-age-dependent-anion channel 1 (VDAC1)/mitochondrial calcium uniporter (MCU) calcium signal axis related genes (*IP3R*, *GRP75*, and *MCU*), mitochondrial function-related genes (peroxisome pro-liferator-activated receptor gamma coactivator 1 alpha (*PGC1α*), mitochondria fission factor (*MFF*), and fission protein 1 (*Fis1*)), fatty acid synthesis (FAS)-related genes (fatty acid synthase (*FASN*), sterol regulatory element binding protein 1 (*SREBP1*), and acetyl-CoA carboxylase (*ACC*)), and fatty acid oxidation (FAO)-related genes (acyl-CoA oxidase 1 (*ACOX1*), carnitine palmitoyltransferase 1 (*CPT1*), and peroxi-some proliferator-activated receptor alpha (*PPARα*)) were measured. Relative gene expression was calculated using the 2^−ΔΔCt^ method [29] and normalized to β-actin levels. The primer sequences are in Table 2.

### 2.10. Western Blot Analysis

Liver tissue was lysed to extract total cellular protein. The 4–20% SDS-PAGE was used to separate proteins. The PVDF membrane was sealed with five percent skim milk and subsequently treated with rabbit anti-Mitofusin 2 (MFN2) antibody (12186-1-AP, Proteintech, Wuhan, China, dilution 1:5000), rabbit anti-VDAC1 antibody (55259-1-AP, Proteintech, Wuhan, China, dilution 1:2000), and rabbit anti-Tubulin β antibody (AP0064, Bioworld, Nanjing, China, dilution 1:5000). The membranes were then treated with anti-rabbit IgG (SA00001-1, Proteintech, Wuhan, China, dilution 1:5000) at ambient temperature for one hour. Blot bands were imaged utilizing the electrochemiluminescence (ECL, Yeasen, Shanghai, China) assay reagent and ChemiScope 6100 (Clinx, Shanghai, China).

### 2.11. Statistical Analysis

The data were presented as mean ± SEM. The SPSS 19.0 software package (SPSS Inc., Chicago, IL, USA) was used for statistical significance analysis. Group comparisons were conducted utilizing the Student’s *t*-test or one-way ANOVA followed by Tukey’s post hoc test. When *p* < 0.05, it was considered statistically significant.

## 3. Results

### 3.1. Effects of CGA on the Performance of Hens Subjected to H_2_O_2_

**Figure 1:**
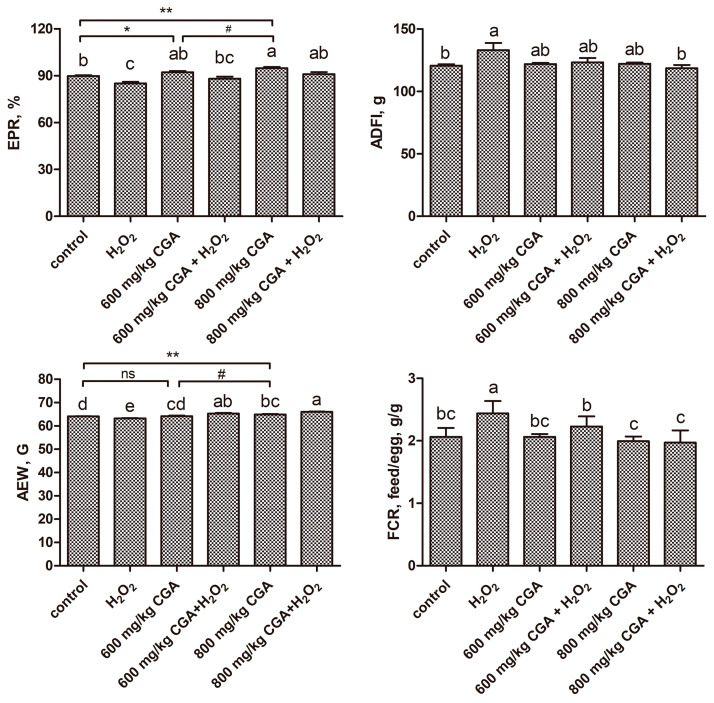
Effects of CGA on the laying performance of hens exposed to H_2_O_2_ (*n* = 6 replicates per treatment and *n* = 60 hens per treatment). ^a–e^ Distinct superscript characters differ significantly (*p* < 0.05). * *p* < 0.05 and ** *p* < 0.01 vs. control (Student’s *t*-test). ^#^ *p* < 0.05 vs. 600 mg/kg CGA group (Student’s *t*-test). ns, nonsignificant (*p* > 0.05). Abbreviation: H_2_O_2_, hydrogen peroxide; CGA, chlorogenic acid; EPR, egg production rate; ADFI, average daily feed intake; AEW, average egg weight; FCR, feed conversation rate.

As presented in Figure 1, when compared to control, 600 and 800 mg/kg CGA treatment significantly enhanced the EPR (89.81 ± 1.66% (control), 92.29 ± 3.54% (600 mg/kg CGA), 94.43 ± 4.13% (800 mg/kg CGA)) (*p* < 0.05). The 800 mg/kg CGA significantly increased AEW (*p* < 0.05). Additionally, when contrasted with the 600 mg/kg CGA group, the 800 mg/kg CGA treatment significantly enhanced both ERP and AEW (*p* < 0.05). Conversely, H_2_O_2_ treatment markedly decreased the EPR and AEW and increased FCR and ADFI versus the control (*p* < 0.05). CGA pretreatment mitigated the adverse impacts of H_2_O_2_ treatment on the EPR, ADFI, AEW, and FCR versus the H_2_O_2_ group.

### 3.2. Effects of CGA on the Egg Quality of Hens Subjected to H_2_O_2_

As shown in Table 3, when contrasted with the control, 600 and 800 mg/kg CGA significantly improved the Haugh unit (HU), albumen height (AH), and yolk color (YC) (*p* < 0.05). Conversely, H_2_O_2_ treatment markedly reduced the HU and AH (*p* < 0.05). When compared with the H_2_O_2_ treatment group, CGA pretreatment significantly improved the HU and AH in laying hens under H_2_O_2_ stress (*p* < 0.05).

### 3.3. Effects of CGA on the Serum Redox Status of Hens Subjected to H_2_O_2_

**Figure 2:**
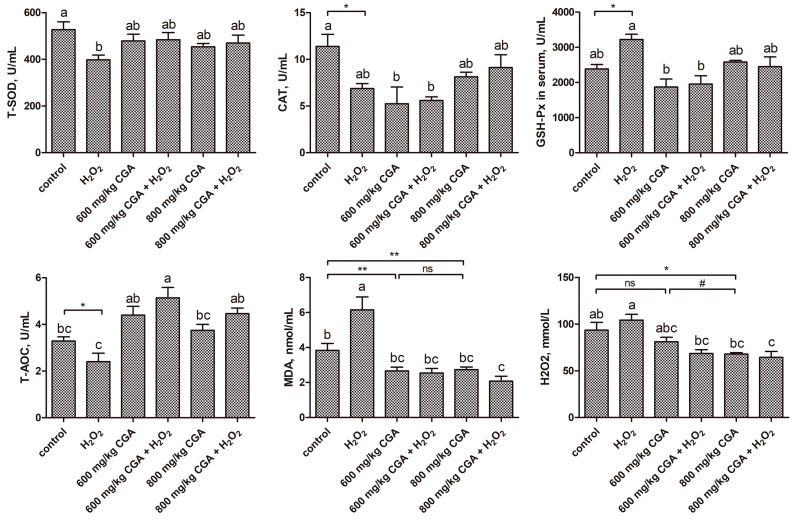
Effects of CGA on the serum redox status of hens exposed to H_2_O_2_ (*n* = 12). ^a–c^ There is a significant difference in the columns with different superscript letters (*p* < 0.05). * *p* < 0.05 and ** *p* < 0.01 vs. control (Student’s *t*-test). ^#^ *p* < 0.05 vs. 600 mg/kg CGA group (Student’s *t*-test). ns, nonsignificant (*p* > 0.05). Abbreviation: H_2_O_2_, hydrogen peroxide; CGA, chlorogenic acid; CAT, catalase; T-SOD, total superoxide dismutase; GSH-Px, glutathione peroxidase; T-AOC, total antioxidant capacity; H_2_O_2_, hydrogen peroxide.

As shown in Figure 2, the 600 mg/kg CGA treatment notably reduced MDA levels, while the 800 mg/kg CGA treatment significantly lowered both MDA and H_2_O_2_ levels compared to the control (*p* < 0.05). Additionally, the 800 mg/kg CGA treatment exhibited a more pronounced inhibition of H_2_O_2_ levels than the 600 mg/kg group. H_2_O_2_ treatment significantly enhanced the GSH-Px activities and MDA levels while concurrently decreasing the T-SOD, CAT, and T-AOC activities versus the control (*p* < 0.05). The 600 and 800 mg/kg CGA pretreatment significantly reduced serum MDA and H_2_O_2_ levels under H_2_O_2_ stress while normalizing T-SOD, GSH-Px, and T-AOC to control values versus the H_2_O_2_ group.

### 3.4. Effects of CGA on the Liver Redox Status of Hens Subjected to H_2_O_2_

**Figure 3:**
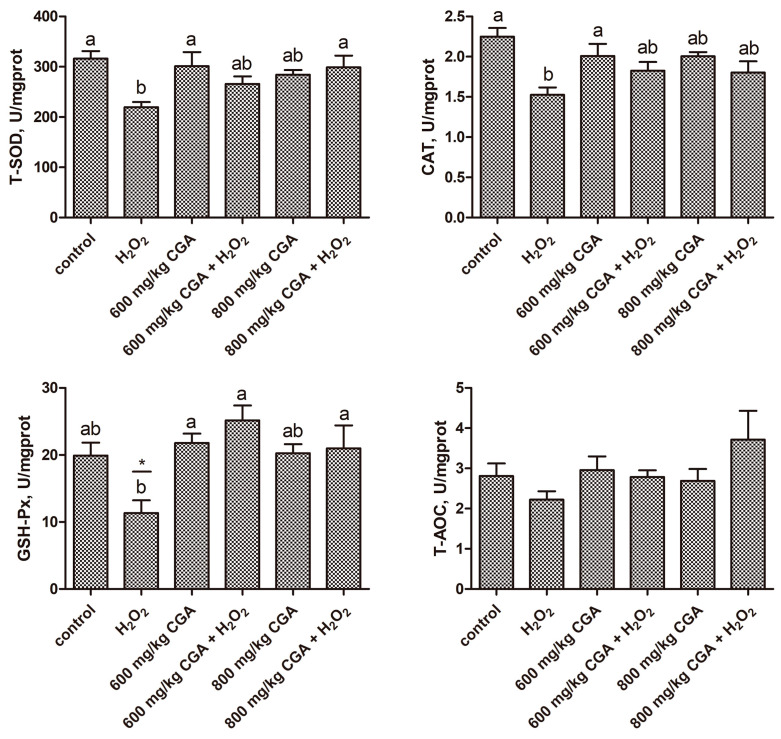
Effects of CGA on liver redox status of hens subjected to H_2_O_2_ (*n* = 12). ^a,b^ There is a significant difference in the columns with different superscript letters (*p* < 0.05). * *p* < 0.05 vs. control (Student’s *t*-test). Abbreviation: H_2_O_2_, hydrogen peroxide; CGA, chlorogenic acid; CAT, catalase; T-SOD, total superoxide dismutase; GSH-Px, glutathione peroxidase; T-AOC, total antioxidant capacity; MDA, malondialdehyde.

As shown in Figure 3, exposure to H_2_O_2_ significantly reduced the CAT, T-SOD, and GSH-Px activities in the liver of hens compared to the control (*p* < 0.05). Conversely, CGA pretreatment normalized hepatic CAT, T-SOD, and GSH-Px levels to control values under H_2_O_2_ stress versus the H_2_O_2_ group.

The results presented above demonstrate that pretreatment with CGA at dosages of both 600 and 800 mg/kg significantly mitigated the reduction in production performance, the deterioration of egg quality, and the antioxidant imbalance induced by H_2_O_2_. Notably, the administration of 800 mg/kg CGA exhibited a superior effect on the laying performance compared to the 600 mg/kg dosage. Consequently, we have opted to utilize the 800 mg/kg CGA for subsequent experimental investigations.

### 3.5. Effects of CGA on Liver Histopathology and Inflammation in Hens Subjected to H_2_O_2_

**Figure 4:**
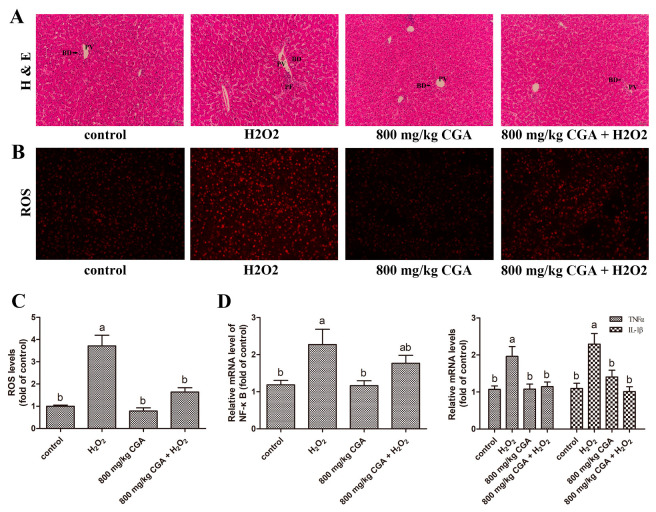
Effects of CGA on liver histopathology and inflammation in hens exposed to H_2_O_2_ (*n* = 12). (**A**) Hematoxylin and eosin stain (H & E, bar = 50 μm). (**B**) ROS staining. (**C**) Quantification of ROS levels in (**B**). (**D**) Real-time PCR detection of *NF-κB*, *TNFα*, and *IL-1β*. ^a,b^ Distinct superscript characters differ significantly (*p* < 0.05). Abbreviations: H_2_O_2_, hydrogen peroxide; BD, bile duct; PV, portal vein; PF, periportal fibrosis; ROS, reactive oxygen species; NF-κB, nuclear factor kappa B; TNFα, tumor necrosis factor-alpha; IL-1β, interleukin-1 beta.

As shown in Figure 4, liver tissues from these H_2_O_2_ treatment groups exhibited periportal fibrosis and periportal inflammatory cell infiltration. Meanwhile, 800 mg/kg CGA treatment significantly alleviated the pathological phenomena of the liver induced by H_2_O_2_ (Figure 4A). In addition, the ROS levels in hepatocytes were detected by immunofluorescence assay, and the findings revealed that H_2_O_2_ treatment significantly elevated the ROS levels versus the control (*p* < 0.05) (Figure 4B,C). The 800 mg/kg CGA pretreatment significantly mitigated H_2_O_2_-induced enhancement in ROS generation versus the H_2_O_2_ group (*p* < 0.05). Further, we evaluated CGA’s effect on the expression of hepatic inflammatory factors under OS. The findings indicated that H_2_O_2_ treatment significantly upregulated *NF-κB*, *TNFα*, and *IL-1β* gene expression compared to the control (*p* < 0.05) (Figure 4D). Pretreatment with 800 mg/kg CGA significantly downregulated the inflammation-related gene expression that was upregulated by H_2_O_2_ exposure versus the H_2_O_2_ group (*p* < 0.05). These data confirmed that OS induced by H_2_O_2_ results in liver injury and inflammation, while pretreatment with CGA can mitigate the OS caused by H_2_O_2_ and alleviate the associated liver injury.

### 3.6. Effects of CGA on Hepatocyte Mitochondrial Function in Hens Subjected to H_2_O_2_

**Figure 5:**
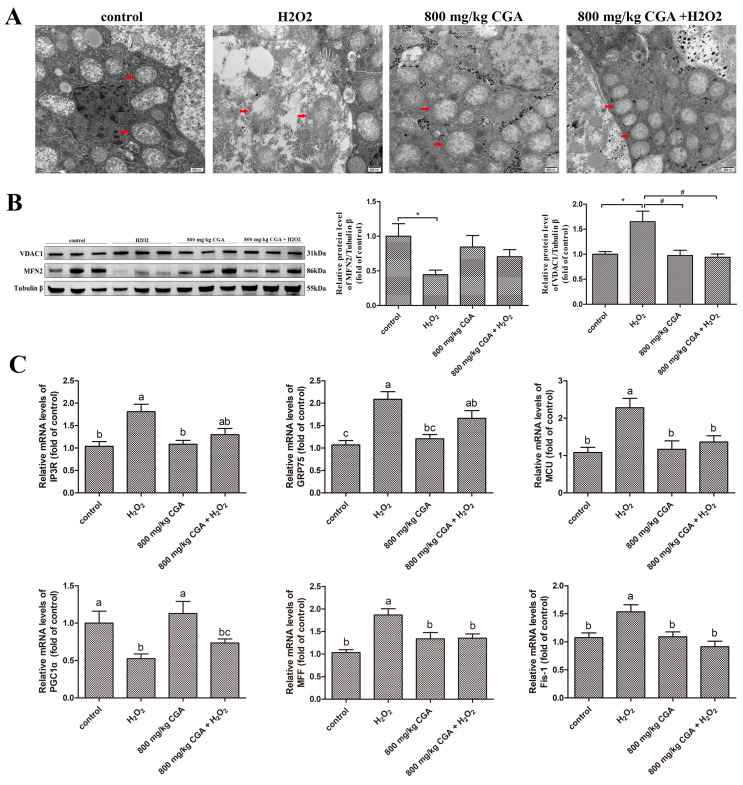
Effects of CGA on the hepatic mitochondrial function of laying hens subjected to H_2_O_2_. (**A**) Mitochondrial structure (imaged by TEM, scale bar: 500 nm, red arrowheads depict mitochondria). (**B**) Western blot detection and quantification of MFN2 and VDAC1 (*n* = 3, equal portions of 4 liver tissues were mixed into one sample). (**C**) Real-time PCR detection of *IP3R*, *GRP75*, *MCU*, *PGC1α*, *Fis1*, and *MFF* (*n* = 12). ^a–c^ There is a significant difference in the columns with different superscript letters (*p* < 0.05). * *p* < 0.05 vs. control (Student’s *t*-test). ^#^ *p* < 0.05 vs. H_2_O_2_ group (Student’s *t*-test). Abbreviations: TEM, transmission electron microscopy; H_2_O_2_, hydrogen peroxide; MFN2, mitofusin 2; IP3R, inositol 1,4,5-trisphosphate receptor; GRP75, glucose-regulated protein 75; VDAC1, voltage-dependent-anion channel 1; MCU, mitochondrial calcium uniporter; PGC1α, Peroxisome proliferator-activated receptor gamma coactivator 1 alpha; MFF, mitochondria fission factor; Fis-1, fission protein 1.

The TEM results demonstrated that the mitochondrial structure of hepatocytes was damaged by H_2_O_2_, and this damage under H_2_O_2_ stress was significantly alleviated in the CGA pretreatment group (Figure 5A). Further, we evaluated changes in the markers associated with mitochondrial function. The data indicated that H_2_O_2_ exposure significantly upregulated the expression of *IP3R*, *GRP75*, *MCU*, and *MFF* genes and VDAC1 protein, and downregulated the expression of *PGC1*α and *Fis1* genes and MFN2 protein compared to the controls (*p* < 0.05). Pretreatment with 800 mg/kg CGA inhibited the downregulation of MFN2 protein, *PGC1α* and *Fis1* genes, and the upregulation of VDAC1 protein, and *IP3R*, *GRP75*, *MCU*, and *MFF* genes by H_2_O_2_ (Figure 5B,C). This suggests that H_2_O_2_ disrupts hepatocyte mitochondrial function by activating the IP3R/GRP75/VDAC1/MCU signaling pathway. CGA provides a protective effect by inhibiting the activation of this pathway under H_2_O_2_-induced stress.

### 3.7. Effects of CGA on Lipid Droplet Levels in Hepatocytes of Hens Subjected to H_2_O_2_

**Figure 6:**
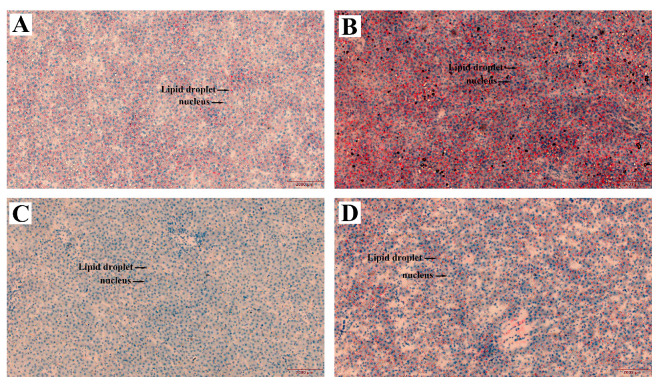
Effects of chlorogenic acid (CGA) on hepatocyte lipid droplet levels in hens subjected to hydrogen peroxide (H_2_O_2_). Oil Red O-stained liver sections (Bar = 2000 μm, *n* = 12) (**A**) control. (**B**) H_2_O_2_. (**C**) 800 mg/kg CGA. (**D**) 800 mg/kg CGA + H_2_O_2_.

Meanwhile, we evaluated the influence of CGA on lipid droplet levels in hepatocytes of hens subjected to H_2_O_2_, with the finding presented in Figure 6. When contrasted with the control, H_2_O_2_ treatment significantly enhanced the liver lipid droplet levels, while 800 mg/kg CGA treatment decreased the liver lipid droplet levels. CGA treatment can alleviate lipid accumulation in hepatocytes caused by H_2_O_2_.

### 3.8. Effects of CGA on Lipid Levels and Lipase Activity in the Serum and Liver of Hens Subjected to H_2_O_2_

As shown in Table 4, the treatment with H_2_O_2_ led to a notable elevation in serum TG and T-CHO levels, as well as an enhancement in the LPL activity compared to the control. Conversely, a significant enhancement in serum HL activity was observed (*p* < 0.05). In the liver, the H_2_O_2_ challenge significantly elevated the TG levels while reducing the T-CHO levels versus the control (*p* < 0.05). The 800 mg/kg CGA treatment notably lowered serum TG, T-CHO, and LDL-C levels while concurrently enhancing LPL activity compared to the control (*p* < 0.05). Furthermore, 800 mg/kg CGA significantly suppressed the elevation of serum TGs, T-CHO, LDL-C, and liver TG contents caused by H_2_O_2_ (*p* < 0.05).

### 3.9. Effects of CGA on Lipid Metabolism-Related Gene Expression in Hens Subjected to H_2_O_2_

**Figure 7:**
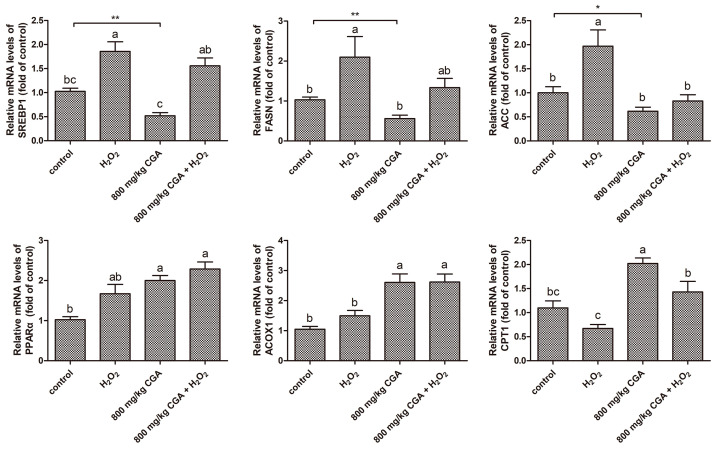
Effects of CGA on liver lipid metabolism gene expression in hens exposed to H_2_O_2_ (*n* = 12). ^a–c^ Distinct superscript characters differ significantly (*p* < 0.05). * *p* < 0.05, ** *p* < 0.01 vs. control (Student’s *t*-test). Abbreviation: H_2_O_2_, hydrogen peroxide; CGA, chlorogenic acid; FASN, fatty acid synthase; SREBP1, sterol regulatory element binding protein 1; ACC, acetyl-CoA carboxylase; ACOX1, acyl-CoA oxidase 1; CPT1, carnitine palmitoyltransferase 1; PPARα, peroxisome proliferator-activated receptor alpha.

As illustrated in Figure 7, when compared to the control, H_2_O_2_ treatment significantly upregulated *FASN*, *SREBP1*, and *ACC* gene expression (*p* < 0.05). The 800 mg/kg CGA significantly downregulated *SREBP1*, *FASN*, and *ACC* gene expression (*p* < 0.05). Furthermore, the 800 mg/kg CGA pretreatment inhibited *SREBP1*, *FASN*, and *ACC* gene expression induced by H_2_O_2_, bringing their levels closer to those of the control group. Additionally, *ACOX1*, *CPT1*, and *PPARα* gene expression levels in the H_2_O_2_ treatment group did not exhibit notable differences compared to the control. However, 800 mg/kg CGA significantly upregulated the expression of these genes under both normal and H_2_O_2_ stress conditions (*p* < 0.05). Together, these data demonstrated that H_2_O_2_ induces lipid accumulation by promoting fatty acid synthesis. In contrast, CGA alleviates liver fat accumulation caused by H_2_O_2_ stress by inhibiting FAS and enhancing FAO.

## 4. Discussion

H_2_O_2_ administration can result in free radical overproduction and acute OS, damaging organ structure and function, inducing a systemic inflammatory response, and ultimately leading to decreased growth performance in animals [30,31,32]. This research indicated that intraperitoneal injection of 10% H_2_O_2_ can significantly reduce the EPR and EW and increase the FCR and ADFI of laying hens. CGA has been widely used in animal production because of its various physiological functions [33,34]. Wen et al. showed that treatment with CGA (stevia extract, isochlorogenic acid content > 50%) significantly increased the EPR of layers [35]. However, some studies have shown that CGA supplementation (250 and 500 mg/kg) has no noticeable effect on the EPR and reproductive performance of aged hens [26]. In this study, 600 and 800 mg/kg CGA supplementation markedly elevated the EPR and/or AEW of laying hens and protected laying hens from the negative effects of H_2_O_2_ on the EPR, EW, FCR, and ADFI. In addition, in this study, H_2_O_2_ treatment reduced egg quality, including HU and albumen height. Pretreatment with 600 and 800 mg/kg CGA significantly improved HU, albumen height, and yolk color while simultaneously protecting them from the toxic effects of H_2_O_2_. Similar results were found upon the incorporation of CGA into the diet of aged hens [26]. Overall, dietary CGA supplementation can improve laying performance and egg quality and enhance the resistance to environmental stress of laying hens.

The continuous ovulation of laying hens during peak periods can easily cause OS. The liver is the primary target of OS, which can be largely attributed to its various biological functions, particularly its role in detoxification [36]. OS is usually the result of overproduction of ROS, impaired antioxidant systems, mitochondrial dysfunction, or a combination of these factors [37]. Research has indicated that OS is a critical element at every stage of liver injury development and has emerged as one of the suitable molecular biomarkers for the in situ identification of liver damage [38]. In this research, H_2_O_2_ treatment significantly decreased serum total antioxidant capacity, increased serum MDA levels, and notably decreased liver CAT, T-SOD, and GSH-Px activities. The results showed that H_2_O_2_ treatment destroyed the antioxidant balance in both the body and liver tissue of layers. Further, we found a noticeable enhancement in ROS accumulation in the liver following exposure to H_2_O_2_. At the same time, pathological liver slides revealed several significant pathological alterations and inflammatory reactions, including periportal vein fibrosis, biliary duct hyperplasia, and inflammatory cell infiltration around the portal veins. Previous research has demonstrated that ROS are crucial in facilitating inflammation and apoptosis [39]. Excessive ROS can initiate proinflammatory signaling, leading to the release of various inflammatory mediators [40]. Thus, we detected the expression status of inflammatory factors in liver tissue and found that intraperitoneal injection of H_2_O_2_ significantly upregulated *NF-κB*, *TNFα*, and *IL-1β* gene expression, which was consistent with pathological observations. Additionally, we observed that CGA pretreatment augmented antioxidant capacity, reduced hepatic ROS levels and expression of inflammatory factors, and significantly alleviated pathological alterations in hepatic tissue. This supports the view that CGA possesses good antioxidant and anti-inflammatory characteristics [25,26]. In summary, CGA pretreatment can effectively preserve the antioxidant balance in both the body and liver, inhibit ROS accumulation and inflammation, and ultimately protect hepatic tissue from OS-induced damage in laying hens subjected to H_2_O_2_.

Mitochondria serve as one of the primary contributors to ROS. Since ROS directly damages mitochondrial enzymes, causes mitochondrial DNA mutation, and alters mitochondrial membrane permeability, mitochondria are especially susceptible to oxidative damage [41]. In this research, H_2_O_2_ treatment induced significant injury to the mitochondrial structure of hepatocytes, notably reducing MFN2 protein and *PGC1α* gene expression while increasing *Fis1* and *MFF* gene expression. PGC1α, a major controller of mitochondrial biogenesis, has been shown to alleviate TNF-α-induced liver ischemia-reperfusion injury by regulating MFN2 [42]. MFN2 is responsible for maintaining MAMS coupling structure and mitochondria fusion. The absence or downregulation of MFN2 can disrupt mitochondria fusion and lead to abnormalities in energy metabolism [43]. Mitochondrial division is mainly controlled by DRP1, Fis1, and MFF, and excessive mitochondrial division leads to reduced mitochondrial quality and dysfunction [44]. The findings demonstrated that H_2_O_2_ exposure damaged mitochondrial dynamics and disrupted normal mitochondrial function in hens’ hepatocytes. Further, we found that H_2_O_2_ exposure significantly enhanced *IP3R*, *GRP75*, *MCU* mRNA, and VDAC1 protein levels in hepatocytes. Research has demonstrated that VDAC, situated in the outer mitochondrial membrane, interacts with IP3R to promote the transfer of Ca^2+^ from the endoplasmic reticulum to the mitochondrial intermembrane space [45]. MCU is an essential channel protein that mediates the entry of Ca^2+^ into the mitochondrial matrix [46]. It is consistent with our previous results, which indicated that H_2_O_2_ treatment can cause mitochondrial Ca^2+^ overload by disrupting the coupling structure of mitochondria and endoplasmic reticulum and activating the IP3R/GRP75/VDAC1/MCU signaling axis, thereby inducing cellular OS [30]. In liver cells exposed to OS, CGA preconditioning upregulated the expressions of *PGC1α* and Mfn2 while downregulating the expression of *Fis-1* and *MFF*. This indicates that CGA is pivotal in regulating mitochondrial homeostasis under OS, which is crucial for maintaining normal mitochondrial morphology and function. Meanwhile, CGA pretreatment significantly inhibited the H_2_O_2_-induced upregulation of *IP3R*, *GRP75*, and *MCU* genes, as well as VDAC1 protein in hepatocytes. These findings indicate that CGA prevents H_2_O_2_-induced mitochondrial ROS generation via the IP3R/GRP75/VDAC1/MCU signaling axis.

The liver lipid metabolism function of layers is often strongly related to production performance, including laying rate, egg weight, yolk color, and HU [19]. Therefore, normal hepatic lipid metabolism is particularly important in peak-laying hens. Studies have confirmed that OS caused by multiple elements can result in increased hepatic lipid accumulation [11,12]. This aligns with our results, which demonstrated that H_2_O_2_ treatment significantly increased hepatic lipid droplet accumulation and elevated TG contents in the liver and both TG and T-CHO contents in the blood. Fatty liver hemorrhagic syndrome (FLHS) is one of the most prevalent metabolic liver disorders in layers, and a prominent characteristic of FLHS is the accumulation of TGs [47]. In liver tissues with FLHS, a high level of oxidative damage was observed [48]. Therefore, reducing cellular ROS levels can help mitigate oxidative damage, which is beneficial for preventing the onset of liver metabolic diseases. In fact, the reduction of OS has been shown to have a lipid-lowering action on liver cells [10]. In this investigation, 800 mg/kg CGA pretreatment notably diminished the increase of liver lipid droplets and TG contents of hens under H_2_O_2_ stress. These results indicate that CGA can maintain the balance of hepatic lipid metabolism in hens by decreasing the occurrence of OS.

Further, we investigated the possible molecular mechanism of CGA alleviating H_2_O_2_-induced hepatic lipid accumulation in hens. This study demonstrated that H_2_O_2_ treatment significantly upregulated liver *SREBP1*, *ACC*, and *FASN* gene expression while downregulated *CPT1* gene expression. In liver cells, FAS is regulated through SREBP-1, which promotes the expression of specific lipogenic factors such as FASN, ACC, and SCD1 [49]. Lipid oxidation is the primary pathway of energy metabolism, which mainly occurs in mitochondria. Research has demonstrated that mitochondrial dysfunction and damage are important contributors to metabolic dysfunction-associated fatty liver disease [50]. CPT1 is a critical gene involved in the process of FAO, functioning as a rate-limiting enzyme in the mitochondrial β-oxidation pathway [51]. These findings imply that H_2_O_2_ treatment facilitates lipid synthesis and inhibits fatty acid oxidative decomposition by inducing mitochondrial dysfunction, thus stimulating lipid accumulation in hepatocytes. In this experiment, 800 mg/kg CGA pretreatment significantly inhibited the increase of FAS-related genes, specifically *SREBP1*, *ACC*, and *FASN*, induced by H_2_O_2_, while promoting the expression of FAO-related genes *ACOX1*, *CPT1*, and *PPARα*. PPARα is prominently expressed in the hepatic tissue and is involved in modulating the expression of CPT1 and ACOX1 (involved in peroxisomal FAO) [52]. These findings indicate that CGA can attenuate OS-induced lipid accumulation in hepatocytes by suppressing FAS and stimulating FAO.

## 5. Conclusions

Dietary supplementation of 600–800 mg/kg CGA can improve laying performance, enhance egg quality, and reduce lipid peroxidation of laying hens. The improvement in laying performance with 800 mg/kg of CGA is superior to that with 600 mg/kg of CGA. This study provides evidence that dietary CGA supplementation can successfully safeguard hens from H_2_O_2_-induced OS by preserving laying performance, egg quality, REDOX status, and liver health. CGA can maintain normal hepatic mitochondrial function through the PGC1α/MFN2 and IP3R/GRP75/VDAC1/MCU signaling axis under OS conditions. CGA alleviates H_2_O_2_-induced liver lipid accumulation by suppressing FAS and activating FAO.

## Figures and Tables

**Table 1:** Components and nutritional composition of the basal diet for hens (as-fed basis).

Items	Value (%)	Nutrient Level ^2^	Value
Corn	61.85	Metabolism energy, MJ/Kg	11.07
Soybean meal (43% CP)	25.00	Crude protein, %	16.33
Fish meal (67% CP)	0.5	Ether extract %	3.23
Soybean oil	0.5	Lysine, %	0.84
Limestone	7.00	Methionine, %	0.36
DL-Met (99.8%)	0.10	Methionine + Cystine, %	0.68
L-Cys (99.0%)	0.05	Available phosphoru, %	0.32
Premix ^1^	5.00	Calcium, %	3.53
Total	100.00		

^1^ Premix provides vitamin A, 8000 IU per kilogram of full-price material; Vitamin D3, 2400 IU; Vitamin E, 12 IU; Vitamin K3, 1.2 mg; Vitamin B1, 1.82 mg; Vitamin B2, 6.2 mg; Vitamin B6, 2.5 mg; Vitamin B12, 0.02 mg; D-biotin, 0.21 mg; Niacin, 28 mg; Folic acid, 0.88 mg; Calcium D-pantothenate, 10 mg; Choline chloride, 400 mg; Calcium hydrogen phosphate, 12 mg; Copper, 10 mg; Manganese, 60 mg; Iron, 60 mg; Iodine, 0.20 mg; Selenium, 0.5 mg. ^2^ Calculated using the Chinese feed database (2015) that contains tables of feed composition and nutritional values in China.

**Table 2:** Genes and primers for qRT-PCR.

Target Gene	Primer Sequence (5′-3′)	Accession No.	Product Size (bp)
*β-actin*	F (Forward): TCCCTGGAGAAGAGCTATGAA	NM_205518.1	113 [30]
	R (Reverse): CAGGACTCCATACCCAAGAAAG		
*NF-κB*	F: CTCCTCAACCTCACTTCCTTAC	NM_001396396.1	205
	R: GCTGTGTGCTTTACCTCTTTG		
*TNFα*	F: ACCACGAGTAGGATGTCTGTA	NM_204267.2	97
	R: CCAGCCACTTTGTGAACTTTG		
*IL-1β*	F: GTCAACATCGCCACCTACAA	NM_204524.2	90
	R: CGGTACATACGAGATGGAAACC		
*IP3R*	F: ACGGCTGCTGAGATTGATAC	XM_046925993.1	274 [30]
	R: CTCTCCAGAATAGCCACACTTC		
*GRP75*	F: AAGAGGCAGGCAGTAACTAATC	NM_001006147.2	394 [30]
	R: CTTCAGACTTGTCCAGACCATAG		
*MCU*	F: TTGGCAGAGTGTGAGAGTGG	XM_046920626.1	196 [30]
	R: AATTCCTCGGTCCTCTGCTT		
*PGC1α*	F: TACAGCAATGAGCCTGCCAA	XM_046916274.1	117 [30]
	R: AGGCAATCCATCCTCATCCAC		
*MFF*	F: ACTCAAAGTGGCTCCTCA	XM_040679333.2	80 [30]
	R: CCTGCATAGTTACACTGG		
*Fis-1*	F: GAAGGGAGTGGCCATGTT	XM_040657193.2	106
	R: GTATTCCTTGAGGCGGTAGTT		
*ACOX1*	F: ACTGAGCTGTGTCTCTTGTATG	XM_015295164.2	301
	R: GCTTCAGGTGTTTGTGGAAAG		
*CPT1*	F: GAGAAGAGTGCAGTGAGAAGAG	XM_015286798.2	379
	R: CCAGCCACAGAAGTAGAGTAAG		
*PPARα*	F: GATGCTGCGTGAAGTGAAATG	XM_046906399.1	328
	R: CTGGTGAAAGGGTGTCTGTTAT		
*FASN*	F: CCTGGAGATGTGGAGTATGTTG	NM_205155.3	274
	R: TCAAGGAGCCATCGTGTAAAG		
*SREBP1*	F: GCCATCGAGTACATCCGCTT	XM_048961548.1	103
	R: GGTCCTTGAGGGACTTGCTC		
*ACC*	F: CTCTCTCTTTGGTCGGGATTG	XM_046929960.1	108
	R: CACTGTTCCATGTGCTCAAATAC		

**Table 3:** Effects of CGA on the egg quality of hens exposed to H_2_O_2_.

Items	Control	H_2_O_2_	600 mg/kg CGA	600 mg/kg CGA + H_2_O_2_	800 mg/kg CGA	800 mg/kg CGA + H_2_O_2_	*p*-Value
HU	88.55 ± 1.48 ^ab^	83.38 ± 1.78 ^b,^*	94.85 ± 1.73 ^a,^*	91.05 ± 1.26 ^a^	94.96 ± 1.96 ^a,^*	92.49 ± 1.70 ^a^	0.000
AH, mm	8.28 ± 0.31 ^bc^	7.25 ± 0.33 ^c,^*	9.73 ± 0.40 ^a,^**	8.58 ± 0.27 ^abc^	9.48 ± 0.42 ^ab,^**	8.87 ± 0.29 ^ab^	0.000
YC	6.07 ± 0.07 ^b^	6.25 ± 0.11 ^ab^	6.36 ± 0.09 ^ab,^*	6.40 ± 0.10 ^ab^	6.63 ± 0.13 ^a,^**	6.60 ± 0.12 ^a^	0.003
ES, kgf/m^2^	5.20 ± 0.13	4.87 ± 0.15	5.33 ± 0.12	5.08 ± 0.10	5.39 ± 0.10	5.20 ± 0.13	0.063

Note: ^a–c^ Data (*n* = 6 replicates per treatment and *n* = 30 eggs per treatment) with distinct superscript characters differ significantly (*p* < 0.05). * *p* < 0.05 and ** *p* < 0.01 vs. control (Student’s *t*-test). Abbreviation: H_2_O_2_, hydrogen peroxide; CGA, chlorogenic acid; HU, Haugh unit; AH, albumen height; YC, yolk color; ES, eggshell strength.

**Table 4:** Effects of CGA on serum and hepatic lipid contents and lipase activity in hens.

Items	Control	H_2_O_2_	800 mg/kg CGA	800 mg/kg CGA + H_2_O_2_	*p*-Value
Serum					
TGs, mmol/L	6.60 ± 0.54 ^a^	8.13 ± 0.30 ^a,^*	4.51 ± 0.56 ^b^	4.29 ± 0.51 ^b^	<0.001
T-CHO, mmol/L	1.54 ± 0.09 ^b^	2.01 ± 0.13 ^a^	1.04 ± 0.07 ^c^	0.86 ± 0.11 ^c^	<0.001
LDL-C, mmol/L	1.17 ± 0.19 ^ab^	1.71 ± 0.30 ^a^	0.63 ± 0.07 ^b^	0.49 ± 0.12 ^b^	0.002
LPL, U/L	1.10 ± 0.15 ^b^	2.33 ± 0.17 ^a^	2.97 ± 0.43 ^a^	2.50 ± 0.41 ^a^	0.005
HL, U/L	2.58 ± 0.06 ^a^	1.13 ± 0.14 ^b^	2.40 ± 0.29 ^a^	2.47 ± 0.40 ^a^	0.001
Liver					
TGs, mmol/gprot	0.52 ± 0.13 ^b^	0.95 ± 0.13 ^a^	0.19 ± 0.01 ^b^	0.38 ± 0.06 ^b^	<0.001
T-CHO, mmol/gprot	0.127 ± 0.01 ^a^	0.091 ± 0.01 ^b^	0.098 ± 0.01 ^ab^	0.095 ± 0.01 ^ab^	0.022
LDL-C, mmol/gprot	0.040 ± 0.002	0.047 ± 0.001	0.038 ± 0.009	0.045 ± 0.007	0.641

Note: ^a,b,c^ Data with (*n* = 12) distinct superscript characters differ significantly (*p* < 0.05). * *p* < 0.05 vs. control (Student’s *t*-test). Abbreviations: H_2_O_2_, hydrogen peroxide; CGA, chlorogenic acid; TGs, triglycerides; T-CHO, total cholesterol; LDL-C, low-density lipoprotein cholesterol; LPL, lipoprotein lipase; HL, hepatic lipase.

## Data Availability

The data that support the findings of this study are available from the corresponding author upon reasonable request.
